# Nemitin, a Novel Map8/Map1s Interacting Protein with Wd40 Repeats

**DOI:** 10.1371/journal.pone.0033094

**Published:** 2012-04-16

**Authors:** Wei Wang, Victor F. Lundin, Ivan Millan, Anne Zeng, Xinyu Chen, Jie Yang, Elizabeth Allen, Ningna Chen, Gillian Bach, Andrew Hsu, Michael T. Maloney, Mridu Kapur, Yanmin Yang

**Affiliations:** 1 Department of Neurology, Stanford University School of Medicine, Stanford, California, United States of America; 2 Department of Biochemistry and Cell Biology, Shanghai Jiao Tong University School of Medicine, Shanghai, China; Institut Curie, France

## Abstract

In neurons, a highly regulated microtubule cytoskeleton is essential for many cellular functions. These include axonal transport, regional specialization and synaptic function. Given the critical roles of microtubule-associated proteins (MAPs) in maintaining and regulating microtubule stability and dynamics, we sought to understand how this regulation is achieved. Here, we identify a novel LisH/WD40 repeat protein, tentatively named nemitin (*n*euronal *e*nriched *M*AP *i*nteracting protein), as a potential regulator of MAP8-associated microtubule function. Based on expression at both the mRNA and protein levels, nemitin is enriched in the nervous system. Its protein expression is detected as early as embryonic day 11 and continues through adulthood. Interestingly, when expressed in non-neuronal cells, nemitin displays a diffuse pattern with puncta, although at the ultrastructural level it localizes along the microtubule network *in vivo* in sciatic nerves. These results suggest that the association of nemitin to microtubules may require an intermediary protein. Indeed, co-expression of nemitin with microtubule-associated protein 8 (MAP8) results in nemitin losing its diffuse pattern, instead decorating microtubules uniformly along with MAP8. Together, these results imply that nemitin may play an important role in regulating the neuronal cytoskeleton through an interaction with MAP8.

## Introduction

The microtubule (MT) cytoskeleton serves as a physical substrate for a wide range of biochemical processes including signal transduction and intracellular transport. It is well established that the dynamics and organization of the microtubule cytoskeleton are regulated largely by microtubule associated proteins (MAPs), particularly in neurons where a specific set of MAPs is expressed. In contrast, there has been relatively little effort to understand the roles of MAPs as adaptor proteins mediating the myriad interactions between microtubules and MT-dependent cellular processes. Recent evidence supports such a role. For example syntabulin appears to associate directly with microtubules and also binds to kinesin and syntaxin vesicles [1]. A deeper understanding of the diversity of MAP function is important in light of growing evidence that aberrant MAP functions can contribute to neurodegeneration [2]–[6].

As abundant decorators of the neuronal microtubule cytoskeleton, MAPs are well-positioned to act as mediators between the microtubule cytoskeleton and the rapidly diffusing cytoplasm. Beyond their roles in regulating the balance between microtubule dynamics and stability, neuronal MAPs have also been proposed to control the higher-order structure of the microtubule cytoskeleton, such as the extensive microtubule bundling that is evident in axons and dendrites. Several MAP-interacting proteins have been identified; MAP1B, for example, interacts with the actin cytoskeleton, signaling molecules, cell surface receptors, and the gigaxonin E3 ubiquitin ligase complex [4], [7]–[9]. Compared to other MAP1-family proteins, MAP8 (also called MAP1S) needs to be further characterized [10], [11]. Pathological accumulation of MAP8 causes excessive microtubule stabilization and disruption of axonal transport [12]. MAP8 has also recently been implicated in autophagy and has been shown to interact with NMDA receptor 3A [13], [14]. The precise function of MAP8 in these various processes remains to be elucidated.

**Figure 1:**
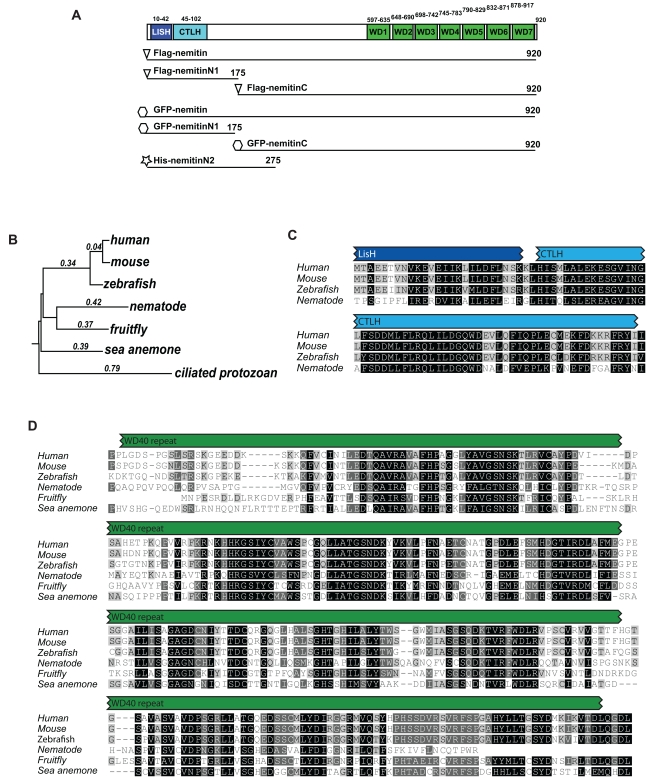
Nemitin domain architecture and phylogenetic distribution. (**A**) Domain diagram of nemitin showing the N-terminal LisH and CTLH domains and the C-terminal WD40 repeat domain. Amino acid numbers shown indicate the locations of predicted features based on mouse nemitin (NP_852065.2). Also shown are schematic representations of the antigen used for antibody production (His-nemitin-N2, indicated by a star), as well as FLAG-tagged and GFP-tagged truncation variants used for interaction studies. (**B**) Phylogenetic tree showing representative species that possess nemitin orthologs as defined by OrthoMCL DB (http://www.orthomcl.org). Common names shown for simplicity, zebrafish = *Danio rerio*, nematode = *Ceanorhabditis elegans*, fruitfly = *Drosophila melanogaster*, sea anemone = *Nematostella vectensis*, ciliated protozoan = *Tetrahymena thermophila*. (**C**) Multiple sequence alignment of the N-terminal domain of nemitin from select sequences. Note that some nemitin sequences without well-conserved LisH/CTLH domains are not included in the alignment. (**D**) Multiple sequence alignment of the WD40 domain of nemitin from select sequences. Note that some species were omitted to prevent ambiguity in the alignment.

Here we identify nemitin (*n*euronal *e*nriched *M*AP *i*nteracting), an uncharacterized protein belonging to the WD40 repeat family. The WD40 repeat domain normally consists of seven WD40 repeats, which together form a seven-bladed β-propeller structure that can support a wide range of protein-protein interactions in a variety of functional contexts [15]. Nemitin also contains a LIS1 homology motif (LisH) and the C-terminal to LisH motif (CTLH) in its amino terminal region (Fig. 1A). LIS1 is a protein which regulates dynein (dynein I/MAP1C) motor activity under high-load transport conditions by interacting with the motor domain and affecting the coordination of multiple dynein complexes [16]. The LisH domain, which mediates oligomerization [17], is the second most common neighbor for the WD40 domain; however, a general functional classification has not been assigned to proteins with this particular domain architecture [15].

Our data show that nemitin is involved in an unknown process linked to the microtubule cytoskeleton. Nemitin is enriched in neurons and localizes to microtubules *in vivo*, yet localizes diffusely or punctate in the cytoplasm when expressed exogenously in cultured non-neuronal cells. We find that nemitin can localize to microtubules through MAP8LC (microtubule-associated protein 8 light chain), since co-expression of MAP8LC caused nemitin to redistribute uniformly along microtubules. Our data provides the first characterization of nemitin, a novel LisH/WD40 protein that associates with microtubules via MAP8 and is likely important for neuronal development and function. Further characterization of nemitin and its interaction with MAP8LC will improve our understanding of MAP8 function and neuronal microtubule regulation.

## Results

### Nemitin is an Evolutionarily Conserved Protein

Since nemitin is a novel uncharacterized protein, we first carried out a phylogenetic analysis to determine its evolutionary conservation among different species. Nemitin is highly conserved among vertebrate species, but is also found in simpler organisms including fly, worm, sea anemone and some single-celled organisms such as the ciliated protozoan *Tetrahymena thermophila* (**Fig. 1B**). Human nemitin contains at least three predicted domains; the lissencephaly-1 homology (LisH) and C-terminal to LisH (CTLH) domains are found in the N-terminus, whereas a seven-repeat WD40 domain is found in the C-terminus (**Fig. 1A**). A multiple sequence alignment shows that all three domains are well conserved in most organisms including vertebrates, zebrafish and *C. elegans* (**Fig. 1C, D**). Notably, human and rodent nemitin are 94% identical, which makes the mouse a suitable model organism for elucidating the function of nemitin. Whereas the WD40 domain has been well-conserved in all nemitin sequences, clearly defined LisH and CTLH domains were not readily detected in some species, including *D. melanogaster*. This suggests that the conserved function(s) of nemitin may depend primarily on the WD40 repeat domain.

### Nemitin is Developmentally Regulated

**Figure 2:**
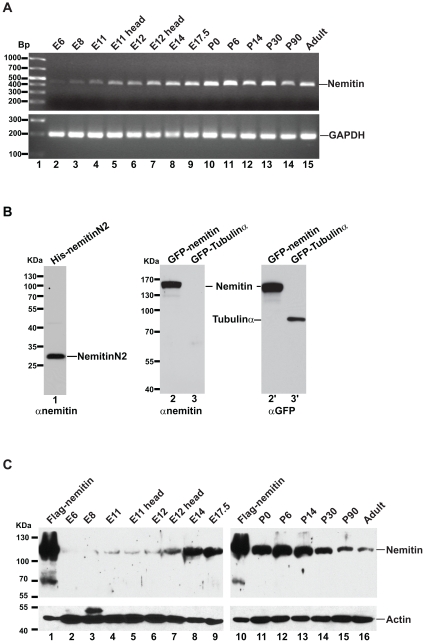
Nemitin’s expression is developmentally regulated. (**A**) Nemitin transcripts were detected as early as E6, increased during subsequent stages of embryonic development and was detected at essentially equal levels in the whole embryo and the head of the embryo at E11 and E12. Expression increased gradually in whole brain tissue of late embryonic and post-embryonic stages and remained essentially constant after P0. (**B**) Rabbit polyclonal antibody characterization. Affinity purified His-nemitin-N2 antiserum specifically recognized both bacteria-purified His-nemitin-N2 and lysate ofcells transfected with GFP-nemitin, but not GFP-tubulin, in a western blot. (**C**) The nemitin antibody recognizes a single band in whole embryo and whole brain lysate from various developmental stages. Nemitin protein was first detected at E11 and continued throughout adulthood, although protein levels after P30 were substantially decreased. mRNA and protein extracts shown in (A) and (C) were prepared from whole embryonic lysates for E6-E12, from dissected heads of E11 and E12 embryos, and dissected whole brain from E14 onward.

To begin to characterize the function(s) of nemitin, we first determined its temporal expression pattern. Developing mouse embryos were collected at several embryonic stages, and lysates were tested for nemitin expression at both mRNA and protein levels (**Fig. 2**). Nemitin transcripts were detected as early as embryonic day 6 (E6), and expression gradually increased from weakly detectable levels to a peak at early post-natal stages (**Fig. 2A**). Nemitin transcript levels in the whole embryo at E11 and E12 were similar to expression in the head of the embryo. We therefore used whole brain tissue to examine the temporal expression in subsequent developmental stages. Nemitin transcript levels did not change drastically after P0. In order to measure the protein expression of nemitin, we developed a polyclonal antibody to His-tagged N-terminal nemitin (His-nemitin-N2, see Methods) (**Fig. 1A**). The affinity-purified antibody reacted specifically to nemitin, recognizing both His-nemitin-N2 and full-length GFP-nemitin, which was also detected with anti-GFP antibody. The GFP-tubulin served as negative control (**Fig. 2B**). The nemitin antibody detects a single band in whole embryo lysates and in whole brain lysates, corresponding to nemitin’s predicted molecular weight of 102 kDa (**Fig. 2C**). The developmental expression pattern of nemitin protein correlated with transcript levels; nemitin was first detected in whole embryo lysate at E11 and strong and increasing protein levels were observed in the brain from E14 to early post-natal development (**Fig. 2C**). Expression continued into adulthood, though at a substantially decreased level. Taken together, these results suggest that nemitin may be especially important during late embryonic and early post-natal development, and that its role in adult mice requires a relatively low but maintained level of protein.

### Nemitin is Enriched in the Nervous System

**Figure 3:**
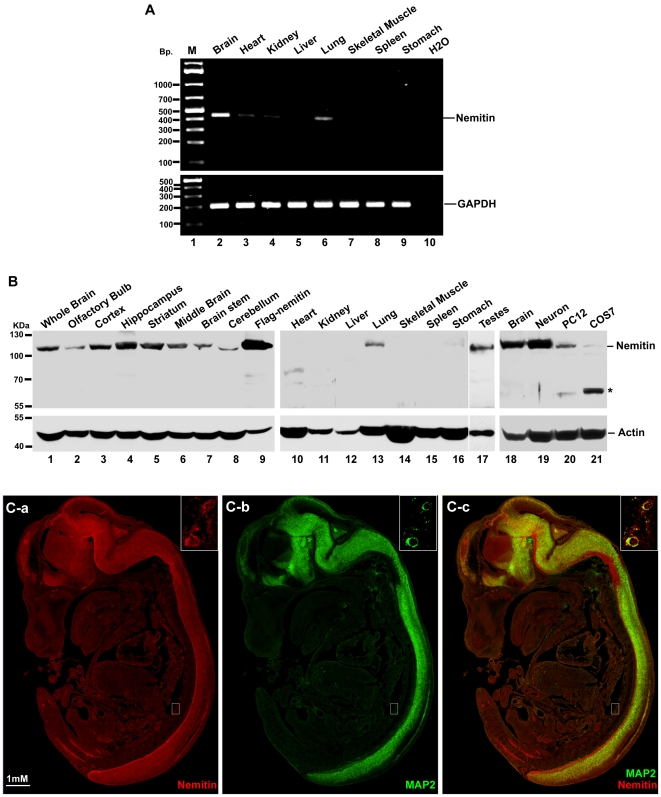
Nemitin is enriched in the nervous system. (**A**) The presence of nemitin transcripts was surveyed in adult mouse tissues by reverse transcriptase-PCR. Nemitin mRNA was observed in the brain, heart, kidney, liver, and lung, with the highest expression in the brain. (**B**) The presence of nemitin protein in adult mouse tissues was visualized by western blot using the nemitin antibody and a β-actin antibody as loading control. Nemitin was found in all brain regions tested (left panel). By contrast, of the other tissues examined, only the lung and testes showed appreciable expression of nemitin (middle panels). Endogenous nemitin protein was detected in cultured cortical neurons as well as the neuronal PC12 cell line, but little or no expression was observed in COS7 cells (right panel). (**C**) Immunostaining of an E14 mouse embryo using nemitin and MAP2 antibodies shows that nemitin is expressed in the brain and spinal cord (C-a) and that its expression correlates closely with MAP2 (C-b). Inset shows appreciable expression of both nemitin and MAP2 in dorsal root ganglia.

We then determined the expression profile of nemitin in adult mice. Nemitin transcripts were most abundant in the brain, but were also detected in heart, kidney and lung tissues (**Fig. 3A**). Significant nemitin protein was found in all brain regions tested (**Fig. 3B**). With the exception of the lung and testes, no other examined tissues were positive for nemitin protein expression (**Fig. 3B** **)**. The differential expression level of nemitin in different brain regions may be attributed to neuronal density or distribution patterns in the area. Furthermore, endogenous nemitin was detected in cultured cortical neurons and in the neuronal PC12 cell line, with weak protein expression in non-neuronal COS7 cells (**Fig. 3B**). Since the tissue distribution of nemitin, as well as its phylogeny, correlates with ciliated cells, we wondered whether nemitin may associate with the cilium. We examined such putative correlation in ciliated IMCD3 cells, and found that nemitin is not enriched in the primary cilium (**Fig. S1**). Taken together, these data show that nemitin is enriched in the brain and suggest that nemitin may be mainly expressed in the nervous system.

To morphologically confirm the expression pattern of nemitin, sagittal sections of whole E14 mouse embryos were stained for nemitin and MAP2. Robust co-localization of nemitin and microtubule-associated protein 2 (MAP2), a MAP enriched in the dendrites of neurons, were observed in the developing brain and spinal cord (**Fig. 3C**), as well as dorsal root ganglia (**Fig. 3C** **, insets**). Significant nemitin staining was also apparent in regions of the developing nervous system where MAP2 staining was absent, suggesting that nemitin may not localize only to dendrites, but also to other cellular compartments such as neuronal axons. These results show that nemitin is expressed in both the central and peripheral nervous systems and that its subcellular distribution likely includes both dendrites and axons.

**Figure 4:**
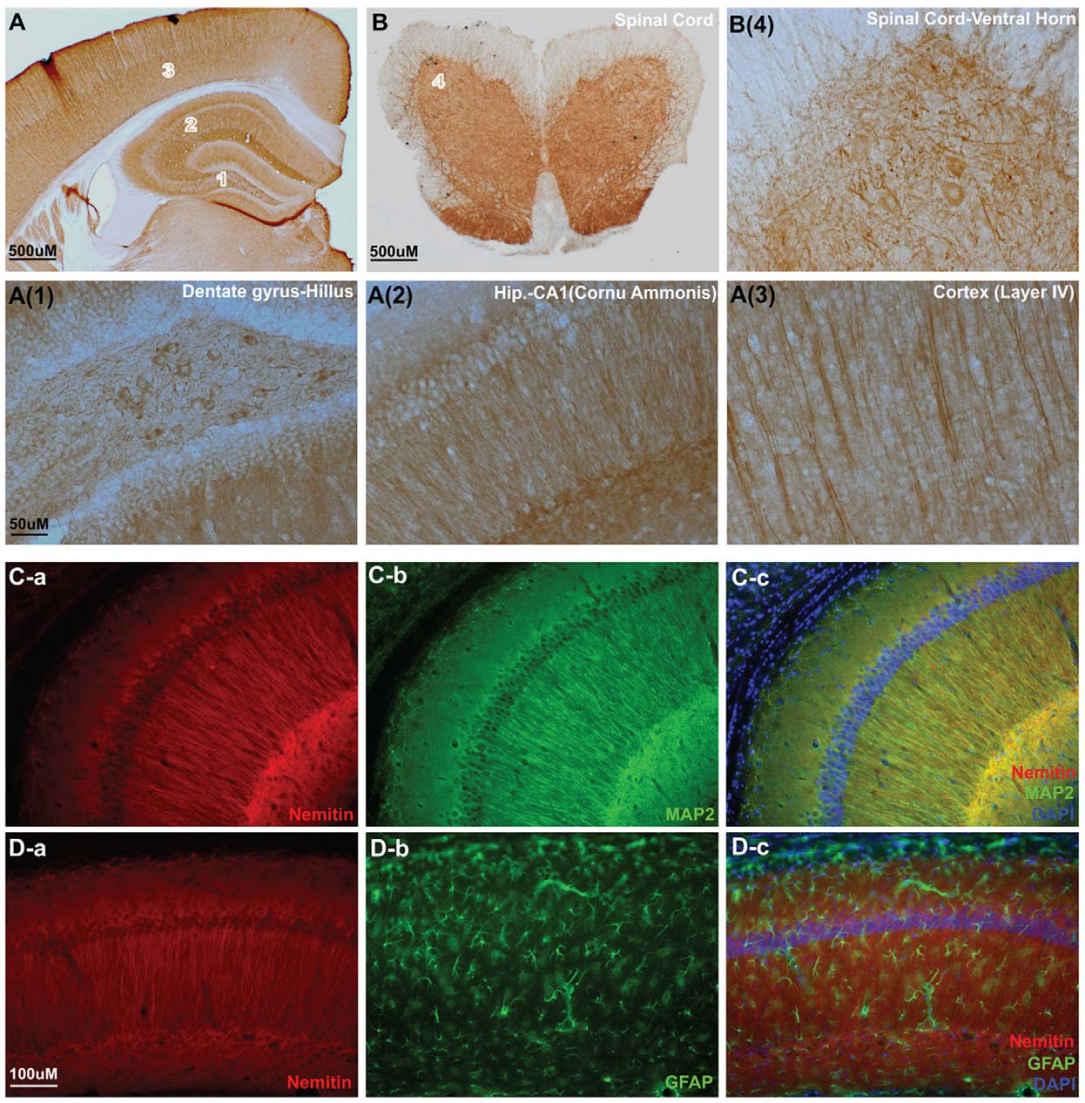
Nemitin is ubiquitously distributed in the nervous system. (**A**) Coronal section of a whole adult mouse brain probed with anti-nemitin antibody and stained with DAB. Expression was evident throughout the brain, especially in the hippocampus (1, 2) and cortex (3). Nemitin staining was visible in cell bodies and fibers in the dentate gyrus (1) and CA1 of the hippocampus (2), as well as fibers in the layer IV of the cortex (3). (**B**) Coronal section of the spinal cord stained as in (A). Nemitin was expressed in the ventral and dorsal grey matter and could be seen in both cell bodies and fibers (4). Some staining was also present in fibers of the white matter. (**C**) Coronal section of CA1 region of adult mouse hippocampus probed with nemitin and MAP2 antibodies showing a close correlation between nemitin and MAP2 expression. (**D**) Coronal section of adult mouse cortex probed with nemitin and GFAP antibodies showing no correlation between nemitin and GFAP expression.

To shed further light on the possible function of nemitin, we characterized the spatial distribution of nemitin in greater detail. Brain and spinal cord sections from adult mice were processed for immunostaining of nemitin. DAB (Diaminobenzidine) staining revealed that nemitin was strongly expressed throughout the cortex and hippocampus (**Fig. 4A**). Similarly, spinal cord grey matter exhibited robust staining for nemitin in both cell bodies and processes (**Fig. 4B**). The neuronal specific localization of nemitin was further confirmed and visualized by double immuno-fluorescence microscopy in mouse hippocampus. We observed nearly a complete co-localization of nemitin with MAP2 (**Fig. 4C**), but not with the astrocyte marker GFAP (**Fig. 4D**). Taken together, these results indicate that within the brain, nemitin is widely expressed and is found more specifically in neurons.

### Nemitin Localizes to Microtubules in Neurons *in vivo*

**Figure 5:**
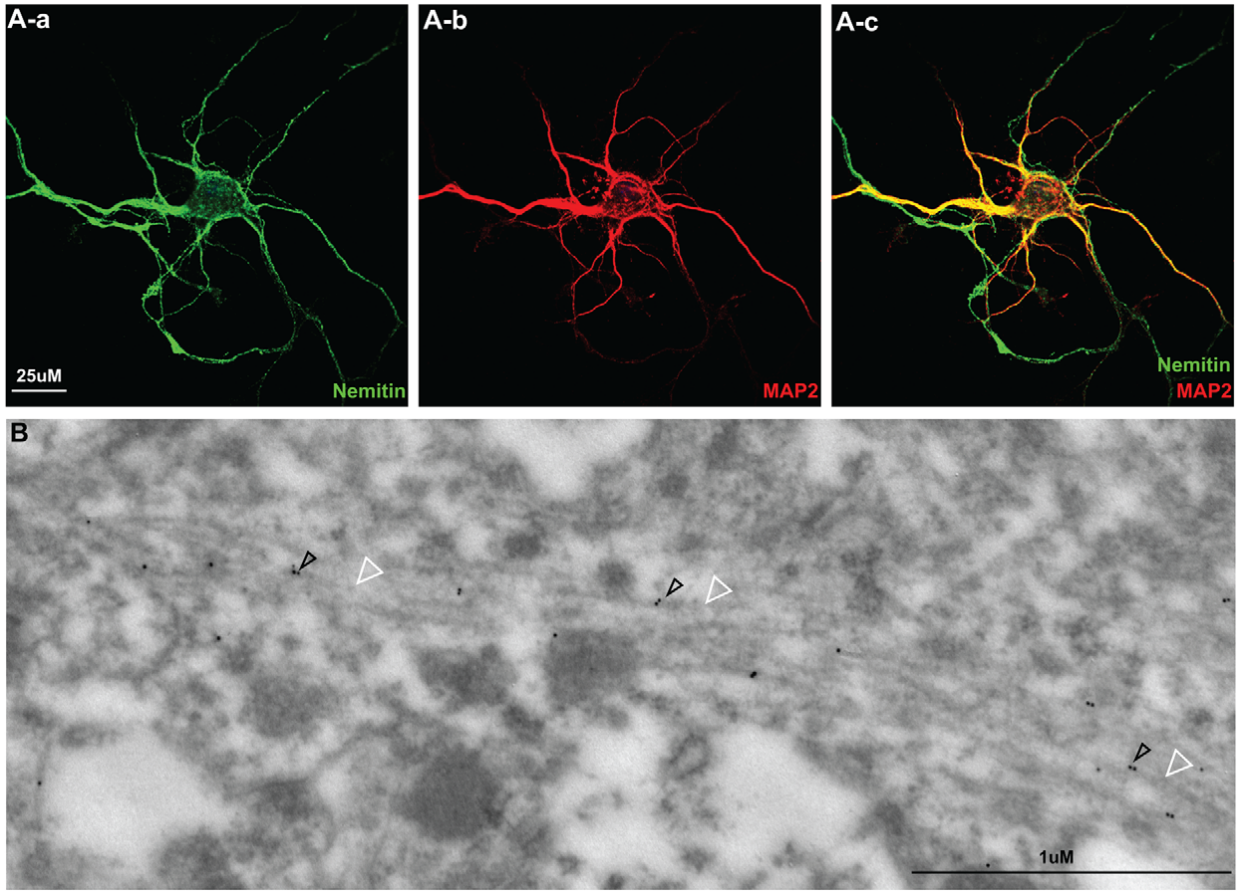
Nemitin localizes to the microtubule cytoskeleton *in vivo*. (**A**) Cortical neurons collected from E15 mouse embryos were cultured 5 DIV and immunostained for endogenous nemitin (a) and MAP2 (b). Nemitin is strongly expressed in both axons and dendrites, whereas MAP2 expression is predominantly dendritic. Further subcellular localization could not be resolved with the fluorescence microscope used here. (**B**) Immuno-gold EM was used to visualize nemitin expression in an adult mouse sciatic nerve section. Nemitin (black arrowheads) is localized precisely along microtubules (white arrowheads) and interspersed throughout the microtubule network.

To further obtain insight into its functional features, we proceeded to investigate the subcellular localization of nemitin in neurons. Cortical neurons were collected from E15 mouse embryos and cultured five days *in vitro*. Staining for endogenous nemitin showed that it was expressed evenly throughout neurons, including the cell body, dendrites, and axons (**Fig. 5A**). In contrast, endogenous MAP2 was preferentially expressed in the cell body and dendrites, but not axons, as previously shown [18]. While MAP2 and nemitin expression overlapped, the limited resolution prevented us from determining exactly where nemitin is expressed within the neuron. To address this, we carried out immuno-gold electron microscopy (EM) in mouse sciatic nerve samples. Approximately 50% of immuno-gold puncta were observed interspersed along microtubules, which strongly suggests that nemitin may be involved in microtubule functions or/and regulations (**Fig. 5B**).

### MAP8LC Mediates Nemitin’s Localization Along Microtubules

**Figure 6:**
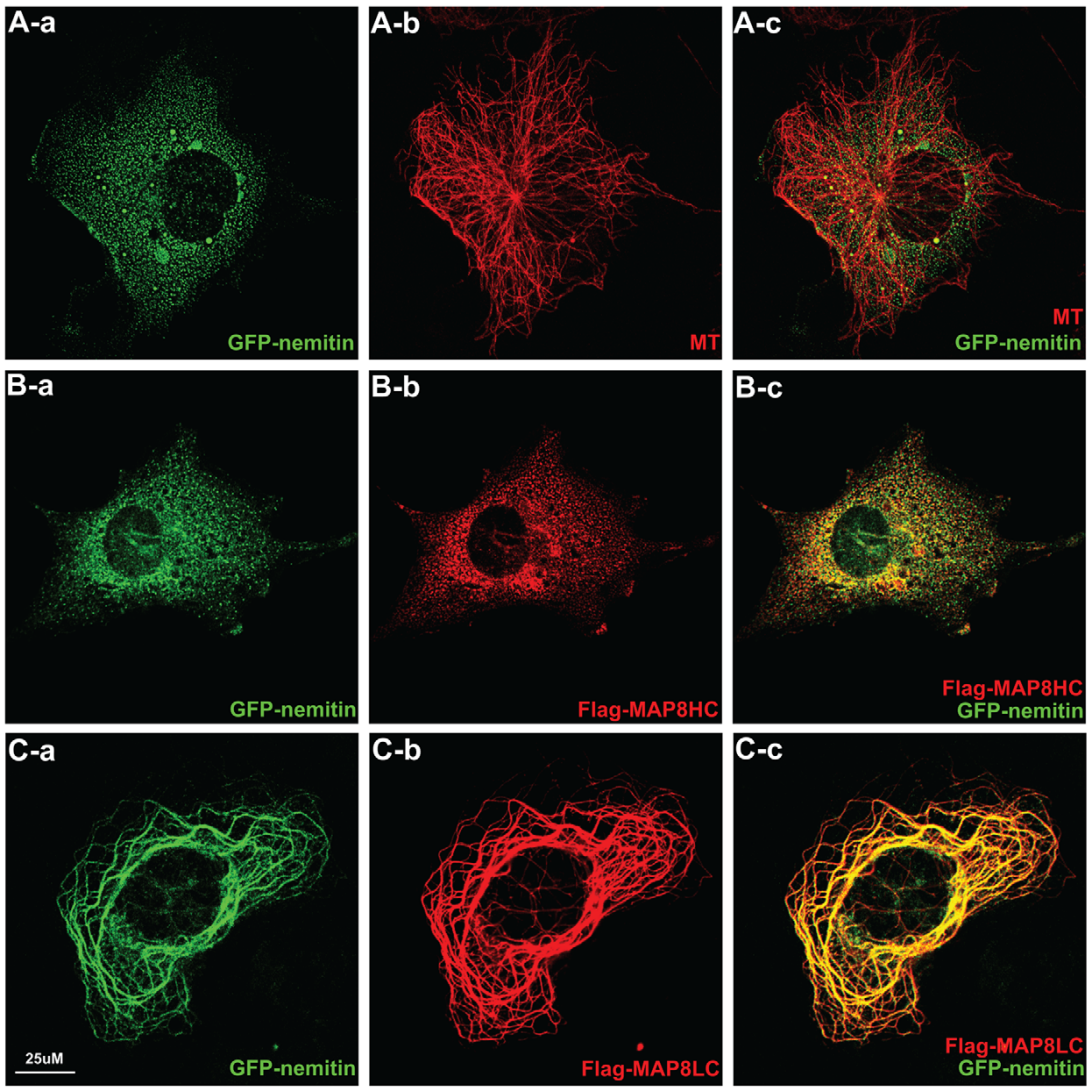
Nemitin localizes with microtubules through MAP8LC. (**A**) COS7 cells were transfected with GFP-nemitin and stained for GFP (a) and alpha-tubulin (b). GFP-nemitin is primarily localized as puncta throughout the cell. A minority of these puncta overlap with the microtubule network (c). (**B**) Cells co-transfected with GFP-nemitin (a) and FLAG-MAP8HC (b) show distinct, non-overlapping patterns (c). (**C**) By contrast, co-transfection of cells with GFP-nemitin and FLAG-MAP8LC results in a complete redistribution of nemitin within the cell. Nemitin fully co-localizes with MAP8LC along the microtubule network.

To understand the association between nemitin and microtubules, we constructed a plasmid encoding human nemitin fused with GFP at its N-terminus (GFP-nemitin) for *in vitro* investigations. Surprisingly, when GFP-nemitin was expressed via transient transfections in COS7 cells, nemitin puncta were diffusely distributed in the cytoplasm without specific microtubule localization (**Fig 6A**). This observation led us to hypothesize that an intermediary protein likely exists in neurons to facilitate the interaction between nemitin and microtubules. We then asked if MAPs, which are abundant in neurons and are required for the regulation of microtubule stability and dynamics, could serve as such an intermediate.

To address this question, we co-transfected COS7 cells with GFP-nemitin and FLAG-MAP8. MAP8 is an important regulator of microtubules in neurons, and we and others have previously defined MAP8/MAP1S as a classic member of the MAP1 family [10]-[12]. Cells expressing both GFP-nemitin and FLAG-MAP8 exhibited a partial co-localization on the microtubule network (data not shown). MAP8 consists of a heavy chain (HC) and light chain (LC). To determine which chain was responsible for this co-localization, COS7 cells were co-transfected with either FLAG-tagged MAP8HC or MAP8LC along with GFP-nemitin. MAP8HC co-expression did not alter the distribution pattern of nemitin (**Fig. 6B**), and the dot-like staining patterns of both MAP8HC and nemitin did not overlap. However, co-transfection of MAP8LC led to a robust relocation of nemitin. Both nemitin and MAP8LC co-localized with the microtubule lattice (**Fig. 6C**). These results indicate that MAP8 plays a crucial role in mediating the association between nemitin and the microtubule network.

### The C-terminal Domain of Nemitin Interacts with the N-terminal Domain of MAP8LC

**Figure 7:**
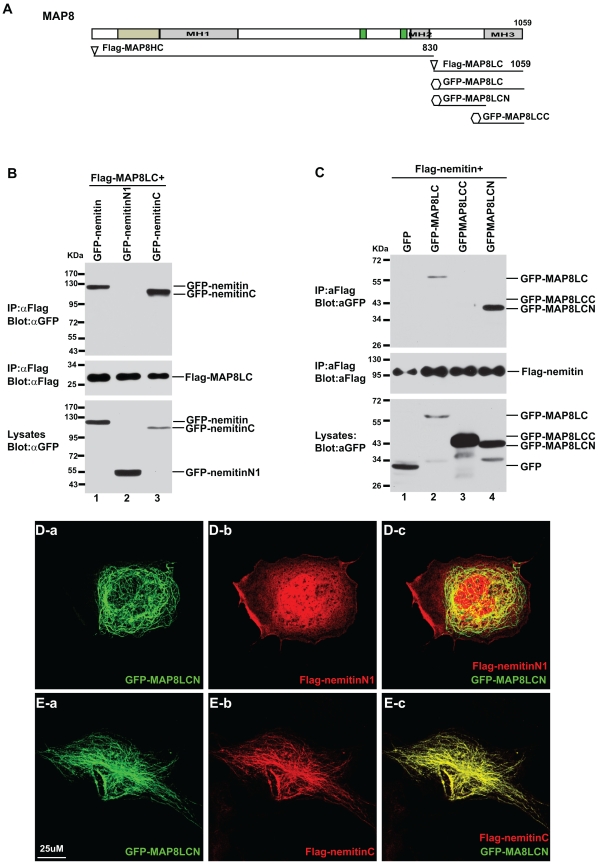
The N-terminal of MAP8LC interacts with the C-terminal of nemitin. (**A**) Schematic representation of MAP8 constructs used in this figure. A similar schematic for nemitin constructs is shown in Figure 1. (**B**) 293T cells were transfected with FLAG-MAP8LC and either full-length, N-terminal, or C-terminal nemitin constructs. Lysates were immunoprecipitated using a FLAG antibody and blotted for the presence of GFP. Both full-length and C-terminal nemitin co-eluted with FLAG-MAP8LC, while N-terminal nemitin was not detected (top panel). As a control, the blot was stripped and reblotted with a FLAG antibody, showing that FLAG-MAP8LC was present (middle panel). Moreover, GFP-nemitin constructs were all present in the initial lysate (bottom panel). (**C**) A similar pulldown experiment determined the region of interaction within MAP8LC. Lysates were immunoprecipated with FLAG-nemitin and blotted for GFP. GFP-MAP8LC and GFP-MAP8LC-N were found in the pulldown, while GFP only and GFP-MAP8LC-C were not. (**D**) COS7 cells were immunostained for GFP-MAP8LC-N (a) and FLAG-nemitin-N (b). While the microtubule network is observed with GFP-MAP8LC-N, FLAG-nemitin has a diffuse pattern, and no co-localization occurs (c). (**E**) Transfection of FLAG-nemitin-C, on the other hand, produced a microtubule pattern and fully co-localized with GFP-MAP8LC-N.

Nemitin contains well conserved LisH-CTLH domain at its N-terminus and WD40 repeats at the C-terminus. We wondered which functional motif, the LisH/CTLH domain or the WD40 repeat module, is responsible for the interaction with MAP8LC. To address this, we used affinity pull-down to determine which nemitin region binds to MAP8LC. 293T cells were co-transfected with FLAG-MAP8LC and a GFP fusion protein containing either full-length, N-terminal (GFP-nemitin-N), or C-terminal nemitin (GFP-nemitin-C). As expected from our staining, the affinity-purified full-length GFP-nemitin co-precipitated with FLAG-MAP8LC (**Fig. 7B**). However, GFP-nemitin-N could not be co-detected with GFP-MAP8LC in immunoprecipitated complex, suggesting that the N-terminal LisH/CTLH region of nemitin doesn’t contain the binding site to MAP8LC. In contrast, the GFP-nemitin-C, which contains the conserved WD40 repeats, was found in the immuno-complex.

While MAP8LC contains multiple binding activities including bindings to microtubules and actins, it can also bind several other proteins [10]–[13]. In order to further dissect the interaction between MAP8LC and nemitin, we determined which sub-region in MAP8LC is responsible for this interaction. Cells were transfected with FLAG-nemitin and GFP-MAP8LC constructs (**Fig. 7A**). FLAG-nemitin pull-down associated with both GFP-MAP8LC and GFP-MAP8LC-N, but not GFP and GFP-MAP8LC-C (**Fig. 7C**). These results reveal that the N-terminal domain of MAP8LC hosts the specific binding sequences to nemitin.

Finally, the domain interactions were further investigated in co-expressed COS7 cells. GFP-MAP8LC-N was co-transfected with either N-terminal or C-terminal of nemitin in COS7 cells. As shown by immunofluorescent microscopy, FLAG-nemitin-N expression alone is diffuse, with no specific cytoskeletal appearance (**Fig. 7D**). However, co-expression caused FLAG-nemitin-C to completely co-localize with MAP8LC-N along the microtubule network (**Fig. 7E**). Thus, we conclude that the C-terminal of nemitin, which includes the conserved WD40 repeat domain, is responsible for MAP8LC-mediated microtubule association.

## Discussion

The microtubule cytoskeleton performs many vital cellular functions, particularly in neurons, and this requires a great diversity of microtubule function which is derived in part from the functions of MAPs. For instance, MAPs have been shown to impact long-range axonal transport, with implications for the pathogenesis of neurodegenerative disease [5], [12]. In this study, we identify and characterize a novel interaction between the light chain of MAP8 and nemitin, a WD40 repeat protein of unknown function. We have shown that nemitin is a neuron-enriched protein that associates with microtubules *in vivo*. However, when expressed in cultured non-neuronal cells, nemitin localized diffusely or in puncta in the cytoplasm. We tested the hypothesis that the microtubule localization of nemitin requires an intermediary protein, showing that nemitin was redistributed to microtubules upon co-expression of MAP8LC. Notably, the spatial and temporal expression pattern of nemitin is closely correlated with MAP8 [11], indicating that these two proteins may interact *in vivo* to regulate the microtubule cytoskeleton or other related cellular processes. Given the finding that nemitin binds MAP8LC, a domain that is highly conserved in several MAP1s, we speculate that nemitin may also interact with other neuronal MAPs, particularly other MAP1 family proteins.

Compared to other members of the MAP1 family, the function of MAP8 is relatively less studied. The light chain of MAP8 decorates and stabilizes microtubules and also contains a functional actin binding site, suggesting that MAP8 might act to crosslink the microtubule and actin cytoskeletal networks [10], [11]. MAP8 accumulation causes excessive microtubule bundling and interferes with axonal transport [12] through an unknown mechanism. Recent evidence also places MAP8 as a linker between microtubules, mitochondria and autophagy. Neonatal MAP8-deficient mice do not have any obvious developmental or behavioral defects, yet display a 3-fold increase in the number of defective mitochondria and a decrease in autophagosome degradation in cardiomyocytes [14]. Since the WD40 domain of numerous proteins acts as a linker in the ubiquitin ligase machinery [19], it may be possible that nemitin plays a similar role in MAP8-mediated authophagy.

It is interesting that nemitin contains the WD40 domain, as several proteins with this motif are associated with neurological disorders. For example, disruption of the LIS1 gene, which also contains a LisH domain and seven WD repeats, leads to lissencephaly [20]. LRRK2 is a WD40 protein associated with Parkinson’s disease, and it has been shown that the WD40 domain is required for neuronal cell death [21]. Intriguingly, the WD40 domain of LRRK2 is also necessary for its association with microtubules [22], similar to our finding that the C-terminal region of nemitin is needed for co-localization with MAP8 along microtubules. The impact of nemitin on MAP8 function in neurons awaits further study, and will likely contribute to a deeper understanding of the role of MAP-interacting proteins in coordinating multiple microtubule-dependent processes.

## Materials and Methods

### Ethics Statement

Guideline in the approved Animal protocol (APLAC #11822, Stanford University School of Medicine) was strictly followed during the procedures involving mouse tissue collections for this study.

Cell linesthat were used in this study areCOS7 (ATCC #CRL-1651), HEK293T (ATCC #CRL-11268), and PC12 (ATCC #CRL-1721), which were originally purchased from ATCC.org and are well established in our laboratory. These cell lines have been used in our previously published studies [4], [23], [24]. IMCD3 cells were a kind gift from Dr. Nachury’s laboratory at Stanford School of Medicine.

### Plasmids

Full length cDNA of nemitin was PCR amplified from human brain cDNA library (Clontech), then sub cloned into mammalian cell expression vector pCDNA3.1D-FLAG and pEGFPC2 (Clontech). Furthermore, the truncation of nemitin N1 and C were amplified with full length nemitin as template, and inserted into pCDNA3.1D-FLAG and pEGFPC2,while the nemitin N2 was amplified and cloned into bacterial expression vector PET28a (Novagen, diagram of nemitin plasmids shown in **Fig.1A**). MAP8HC (heavy chain) and MAP8LC (light chain) in different vectors were described previously [11]. The N and C terminal of MAP8LC were PCR amplified and inserted into pEGFPC2 (diagram of MAP8 plasmids shown in **Fig.7A**). Mammalian cell expression construct pEGFPC2-Tubulin was kindly provided by Dr James Nelson laboratory from Stanford University.

### Phylogenetic Analysis and Multiple Sequence Alignment

The phylogenetic analysis was carried out by querying the Ortho-MCL database (http://www.orthomcl.org) for sequences with similarity to human nemitin isoform 3(NP_001136023.1) (OrthoMCL group accession # OG5_132217). Nemitin sequences retrieved from OrthoMCL DB were aligned and phylogenetic trees were built using Geneious software (Biomatters Ltd). A well-aligned segment of a multiple sequence alignment encompassing the central 258 amino acids of the WD40 domain was used to build a phylogenetic tree of nemitin (**Fig. 1B**). The tree and multiple sequence alignment shown (**Fig. 1C, D**) contain representative species for clarity.

### Preparation of RNA Extracts and Reverse Transcriptase PCR

Total RNA was extracted from embryonic (at the stages indicated in **Fig. 2**) and adult (P90) tissue homogenates using Trizol reagent (Invitrogen) and cDNA was synthesized using oligo(dT)_20_ primer and SuperScript III reverse transcriptase (Invitrogen) according to manufacturer instructions. Primers used for PCR of nemitin: GGACCCCAGTGGCCGTCTCT(forward); GCTCTTTCTGGGGCAGGACGC(reverse); and GAPDH: CACCTTCGATGCCGGGGCTG(forward); TGTTGGGGGCCGAGTTGGGA(reverse).

### Preparation of Protein Extracts

Adult (P90) and embryonic tissues were homogenized in ice-cold extraction buffer (50 mM Tris pH 7.5, 150 mM NaCl, 2 mM EDTA, 1% Triton X-100, 0.5% sodium deoxycholate, 1% SDS) supplemented with complete protease inhibitor cocktail (Roche) using a Dounce homogenizer. Tissue lysates were sonicated to shear genomic DNA and briefly centrifuged to remove cellular debris. Protein concentrations were determined using BCA reagent (Pierce). For developmental expression of nemitin, approximately 200 micrograms of total protein were analyzed by western blotting in order to detect a potentially low abundance of nemitin protein at early embryonic stages. For distribution of nemitin in adult tissues, 80 micrograms of total protein were analyzed, except for testes, for which 125 micrograms were analyzed.

### Cell Culture, Transfection and Immunoprecipitation

Dissociated mouse cortical neurons were cultured in Neurobasal medium supplemented with B27 and Glutamax. PC12 cells were cultured in DMEM with 10% horse serum and 5% FBS. HEK293T and COS7 cells were cultured in DMEM, 10% FBS. IMCD3 cells were cultured in 1∶1 DMEM:F12K media (+glutamine), 10% FBS, and 1% Pen-Strep. To induce cilia, cells were serum starved (0.1% FBS) for 4 hours. Cells were transfected using Calfectin reagent (SignaGen Labs) according to manufacturer instructions. Cells were lysed in ice-cold lysis buffer (50 mM Tris pH 8.0, 500 mM NaCl, 0.5% Triton X-100, 1mM DTT) supplemented with complete protease inhibitor (Roche), briefly sonicated to shear genomic DNA and then centrifuged at 14,000 rpm for 15 minutes at 4°C. For co-immunoprecipitations, soluble lysates were pre-cleared by incubation with protein-A sepharose beads (Rockland) for 30 minutes followed by precipitation with anti-FLAG-M2-agarose beads (Sigma) according to manufacturer instructions.

### Antibodies and Western Blotting

Recombinant nemitin was cloned from a human cDNA library (see Plasmids) to generate His-nemitin-N2 (**Fig. 1A**). Protein was expressed in bacteria, purified, and used for the generation of antibodies. Crude rabbit polyclonal antiserum directed against His-nemitin-N2was obtained from Chemicon (Millipore) and affinity purified. Proteins separated by SDS-PAGE were transferred to PVDF and probed with the following antibodies: 1∶2000 affinity-purified rabbit anti-nemitin, 1∶4000 mouse anti-actin (Sigma), 1∶1000 mouse anti-FLAG (Sigma), 1∶2000 mouse anti-alpha-tubulin (Developmental Studies Hybridoma Bank, 12G10) or 1∶1000 mouse anti-GFP (Santa Cruz Bio.).

### Preparation of Fixed Tissues and Immunostaining

For E14 whole embryo staining, sagittal sections (near the midline, 7-10 micron thickness) were obtained from Zyagen. For adult brain and spinal cord sections, mice were sacrificed at 6 months and perfused with 0.9% heparinized saline (10 U/mL). The whole brain and spinal cord were fixed in 4% PFA for 24hr, rinsed with PBS, and sunk in 30% sucrose in PBS. Coronal brain sections and coronal spinal cord sections were cut at 40 microns with a freezing sliding microtome (Microm HM430) into 16 sequential tubes, so that each tube contained every 16th section, and stored in cryoprotective medium at -20°C. For immunohistochemical detection of nemitin, brain and spinal cord sections were incubated with 1∶300 rabbit anti-nemitin. Labeling was detected with biotinylated goat-anti-rabbit secondary antibody and the Vectastain Elite avidin–biotin complex kit (Vector Laboratories).

### Immunofluoresence of Tissues and Cells

E14 whole embryo sections or adult brain coronal sections were blocked with 3% donkey serum in PBS, incubated with 1∶300 rabbit anti-nemitin and either 1∶1500 mouse anti-MAP2 (Sigma) or 1∶1500 mouse anti-GFAP (Millipore) for 24 hours at 4°C, washed with PBS and incubated with 1∶400 donkey-anti-rabbit Cy3 and 1∶400 donkey-anti-mouse FITC for 24 hours at 4°C. Sections were then washed and mounted in Prolong Anti-fade (Molecular Probes) with DAPI. For subcellular localization of nemitin, cells were washed briefly in PBS and fixed with 100% methanol for 10 minutes at -20°C or 4% PFA for 15 minutes at room temperature, blocked with 1% BSA in PBS, incubated with either 1∶300 rabbit anti-nemitin, mouse anti-α?Tubulin (Developmental Studies Hybridoma Bank, 12G10) and/or mouse anti-FLAG (Sigma) for 24 hours at 4°C, washed with PBS and incubated with either goat-anti-rabbit Alexa 594 and/or goat-anti-mouse Alexa 488 for 1 hour at room temperature. Cells were then washed and mounted in Prolong Anti-fade. For nemitin expression in cilia, IMCD3 cells were incubated with 1∶600 mouse anti-acetylated tubulin (Sigma T7451).

### Immuno-gold Electron Microscopy

Animals were anesthetized, killed, and intravenously perfused with 2% paraformaldehydeand 0.05% glutaraldehyde. The dissected samples of sciatic nerves wereprocessed and embedded for EM [7]. The antibody incorporations of rabbit anti nemitin on ultrathin sections were visualized with 6nm anti–rabbit gold-conjugated particles. After staining with uranyl acetate, followed by lead citrate, the sections were analyzed under a PhilipsCM10 microscope.

### Microscope Image Acquisition

Embryo staining section was scanned with Olympus VS120-SL system. Histology data was captured with ZEISS IMAGER-A1 AX10 and immunofluorescent images were taken using a Leica CTR5000 or TCS SPE DM5500Q.

## Supporting Information

Figure S1 **Nemitin is not enriched in cilia.** Nemitin expression does not specifically localize with cilia. IMCD3 cells were serum starved for 4 hours before fixation to induce outgrowth of primary cilia. (**A**) Cells were stained for endogenous nemitin, which has a dot-like expression pattern. (**B**) Staining for acetylated tubulin identifies cilia (arrow), which are enriched with acetylated tubulin. A portion of the microtubule cytoskeleton is also stained. (**C**) Merge of (A) and (B) showing no specific co-localization of nemitin and cilia.(**D**) DAPI staining merged with (C) shows that the cell nuclei are healthy.
(TIF)
