# Supplementary figures and images for: Nemitin, a Novel Map8/Map1s Interacting Protein with Wd40 Repeats
Source: PLoS One. 2012 Apr 16;7(4):e33094. doi: 10.1371/journal.pone.0033094 (PMC3327699; doi:10.1371/journal.pone.0033094)

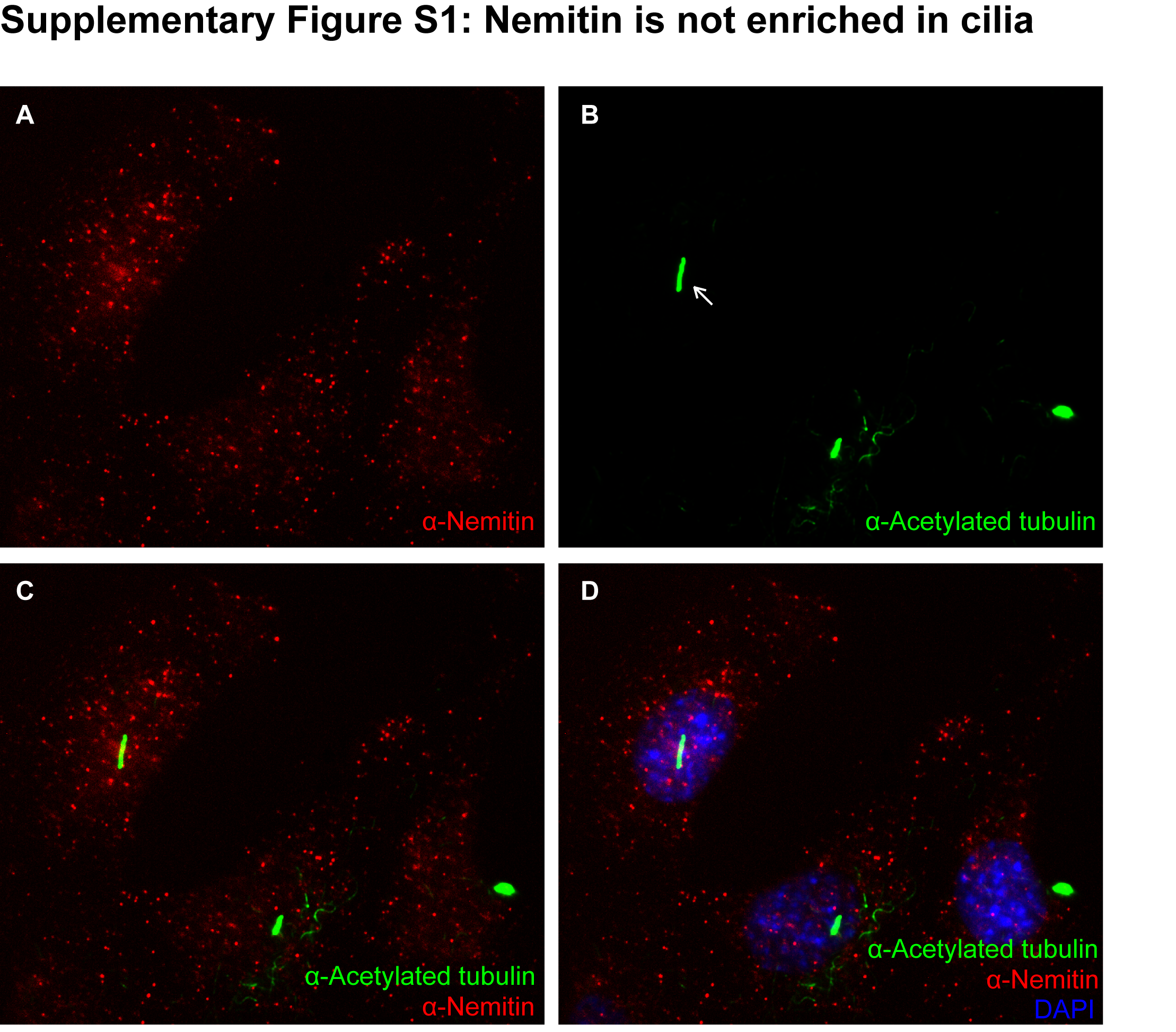

Supplement: Figure S1 — Nemitin is not enriched in cilia. Nemitin expression does not specifically localize with cilia. IMCD3 cells were serum starved for 4 hours before fixation to induce outgrowth of primary cilia. (A) Cells were stained for endogenous nemitin, which has a dot-like expression pattern. (B) Staining for acetylated tubulin identifies cilia (arrow), which are enriched with acetylated tubulin. A portion of the microtubule cytoskeleton is also stained. (C) Merge of (A) and (B) showing no specific co-localization of nemitin and cilia.(D) DAPI staining merged with (C) shows that the cell nuclei are healthy. (TIF) [file pone.0033094.s001.tif]
